# Efficient Genotyping of *KRAS* Mutant Non-Small Cell Lung Cancer Using a Multiplexed Droplet Digital PCR Approach

**DOI:** 10.1371/journal.pone.0139074

**Published:** 2015-09-28

**Authors:** Alexandra Pender, Isaac Garcia-Murillas, Sareena Rana, Rosalind J. Cutts, Gavin Kelly, Kerry Fenwick, Iwanka Kozarewa, David Gonzalez de Castro, Jaishree Bhosle, Mary O’Brien, Nicholas C. Turner, Sanjay Popat, Julian Downward

**Affiliations:** 1 Lung Cancer Group, Division of Molecular Pathology, The Institute of Cancer Research, London, United Kingdom; 2 Department of Medicine, The Royal Marsden NHS Foundation Trust, London, United Kingdom; 3 The Francis Crick Institute, London, United Kingdom; 4 The Breakthrough Breast Cancer Research Centre, The Institute of Cancer Research, London, United Kingdom; 5 The Centre for Molecular Pathology, The Institute of Cancer Research, Sutton, United Kingdom; UNIVERSITY MAGNA GRAECIA, ITALY

## Abstract

Droplet digital PCR (ddPCR) can be used to detect low frequency mutations in oncogene-driven lung cancer. The range of *KRAS* point mutations observed in NSCLC necessitates a multiplex approach to efficient mutation detection in circulating DNA. Here we report the design and optimisation of three discriminatory ddPCR multiplex assays investigating nine different *KRAS* mutations using PrimePCR™ ddPCR™ Mutation Assays and the Bio-Rad QX100 system. Together these mutations account for 95% of the nucleotide changes found in *KRAS* in human cancer. Multiplex reactions were optimised on genomic DNA extracted from *KRAS* mutant cell lines and tested on DNA extracted from fixed tumour tissue from a cohort of lung cancer patients without prior knowledge of the specific *KRAS* genotype. The multiplex ddPCR assays had a limit of detection of better than 1 mutant *KRAS* molecule in 2,000 wild-type *KRAS* molecules, which compared favourably with a limit of detection of 1 in 50 for next generation sequencing and 1 in 10 for Sanger sequencing. Multiplex ddPCR assays thus provide a highly efficient methodology to identify *KRAS* mutations in lung adenocarcinoma.

## Introduction

Lung cancer is the leading cause of cancer-related mortality worldwide [1] and over 20 000 cases of non-small cell lung cancer (NSCLC) were diagnosed in the UK in 2012 [2]. The most frequently mutated oncogenes in lung adenocarcinoma are the *RAS* family GTPases and *EGFR* (25% and 15% respectively [3]). Knowledge of the molecular profile of advanced lung adenocarcinoma is critical for therapeutic decision-making [4], particularly in the use of EGFR and ALK tyrosine kinase inhibitors [5,6]. The presence of a *KRAS* mutation may also be of therapeutic relevance if MEK inhibitor and taxane combinations prove efficacious in this patient cohort [7]. Obtaining adequate tumour tissue for conclusive genotyping in lung cancer can be problematic, however [8]. Droplet digital PCR (ddPCR) is a sensitive method of quantitative mutation detection [9,10] that has the potential to accurately genotype patient-derived material from a small amount of starting material.

Detection of *KRAS* hotspot mutations by ddPCR has been limited by the variety of potential alleles within adjacent loci, although some *KRAS* mutations occur more commonly in cancer than others (Tables 1 and 2). The four commonest mutations account for 80% of all KRAS nucleotide changes found in human cancers (85% of all changes in NSCLC), while the nine commonest mutations account for 95% of all changes and 97.5% of changes in NSCLC. Fluorophore—labelled digital PCR probes complement a particular mutant DNA sequence and so only detect one specific *KRAS* mutation. Using these assays in duplex with a probe to detect the mutant allele and a probe to detect the wild-type allele allow mutant allele fraction calculation for a given mutation, but this approach requires potentially multiple assays and the use of more material before correct identification of the genotype. Development of a multiplex assay combining several different mutant probes in the same reaction is therefore an attractive alternative. Multiplex *KRAS* digital PCR assays have been described using the RainDrop™ Digital PCR system (RainDance Technologies, Billerica, Massachusetts, USA) in advanced colorectal cancer [11] but not as yet with the Bio-Rad QX100 system, an affordable digital PCR system with commercially-available probes for several *KRAS* mutations, nor has a multiplex tool been used in lung cancer.

**Table 1:** *KRAS* mutations observed in human cancer in order of decreasing frequency (http://www.sanger.co.uk/cosmic; accessed 14^th^ July 2015).

KRAS genotype	Mutation	Cases	Frequency
G12D	c.35G>A	10719	33.8%
G12V	c.35G>T	7138	22.5%
G13D	c.38G>A	3959	12.5%
G12C	c.34G>T	3713	11.7%
G12A	c.35G>C	1694	5.3%
G12S	c.34G>A	1507	4.7%
G12R	c.34G>C	1024	3.2%
G13C	c.37G>T	276	0.9%
Q61H	c.183A>C	144	0.5%

**Table 2:** *KRAS* mutations observed in human NSCLC in order of decreasing frequency (http://www.sanger.co.uk/cosmic; accessed 14^th^ July 2015).

KRAS genotype	Mutation	Cases	Frequency
G12C	c.34G>T	604	36.7%
G12V	c.35G>T	350	21.3%
G12D	c.35G>A	336	20.4%
G12A	c.35G>C	107	6.5%
G13C	c.37G>T	54	3.3%
G12S	c.34G>A	53	3.2%
G13D	c.38G>A	43	2.6%
G12R	c.34G>C	36	2.2%
Q61H	c.183A>C	20	1.2%

We set out to design multiplex digital PCR assays which would accurately identify nine different *KRAS* mutations and to demonstrate the application of these assays to patient-derived material using the Bio-Rad QX100 system.

## Materials and Methods

### Digital PCR probe assays

Each digital PCR probe is an oligonucleotide specific to the region of interest with a 5’ fluorophore and a 3’ quencher. The fluorophore is either HEX for probes specific to wild-type sequences or FAM for mutant probes. *KRAS* c.35G>T (G12V; cat no. dHsaCP2500592), c.35G>A (G12D; cat no. dHsaCP2500596), c.35G>C (G12A; cat no. dHsaCP2500586), c.34G>A (G12S; cat no. dHsaCP2500588), c.34G>C (G12R; cat no. dHsaCP2500590), c.34G>T (G12C; dHsaCP2500584), c.37G>T (G13C; cat no. dHsaCP2500595), c.38G>A (G13D; cat no. dHsaCP2500598), c.183A>C (Q61H; cat no. dHsaCP2000133), WT for c.35G>T (WT for G12V; cat no. dHsaCP2500593), WT for c.35G>A (WT for G12D; cat no. dHsaCP2000002), WT for c.35G>C (WT for G12A; cat no. dHsaCP2000004), WT for c.34G>A (WT for G12S; cat no. dHsaCP2000012), WT for c.34G>C (WT for G12R; cat no. dHsaCP2000010), WT for c.34G>T (WT for G12C; cat no. dHsaCP2000008), WT for c.37G>T (WT for G13C; cat no. dHsaCP2500595), WT for c.38G>A (WT for G13D; cat no. dHsaCP2000014) and WT for c.183A>C (WT for Q61H; cat no. dHsaCP2000132) 20x PrimePCR™ ddPCR™ Mutation Assays (containing both primers and ddPCR probes) were purchased from Bio-Rad (Bio-Rad Laboratories, Hercules, California, USA) and used in this study.

### Assay optimisation and DNA quantification

Digital PCR assays were optimised using cell line genomic DNA (gDNA) or synthetic oligonucleotides as appropriate. Cell lines used for this study were authenticated using STR profiling by the Cell Services facility at the Cancer Research UK London Research Institute and the Institute of Cancer Research. These included NCI-H727 (*KRAS* G12V, lung), NCI-H358 (*KRAS* G12C, lung), A549 (*KRAS* G12S, lung), SK-LU-1 (*KRAS* G12D, lung), A427 (*KRAS* G12D, lung), HCT-116 (*KRAS* G13D, colon) and NCI-H1975 (*KRAS* WT, lung). All cell lines were provided directly by Cancer Research UK Cell Services with the exception of HCT-116 (American Type Culture Collection, Rockville, Maryland, USA; cat. no ATCC^®^CCL-247^TM^). Cell gDNA was extracted using the DNeasy™ Blood and Tissue Kit (Qiagen, Venlo, Limburg, Netherlands) as per manufacturer’s instructions. All DNA used in subsequent digital PCR reactions, except synthetic oligonucleotides, was quantified on a QX100 ddPCR system using human RNase P TaqMan® Copy Number Reference Assay (Life Technologies Corporation, Carlsbad, California, USA). 1 μL of eluate was added to a digital PCR reaction containing 10 μL ddPCR Supermix for probes (Bio-Rad) and 1 μL of TaqMan Copy Number Reference Assay, human, RNase P (Life Technologies) on a total volume of 20 μL. The reaction was partitioned into ~14,000 droplets per sample in a QX-100 droplet generator according to manufacturer’s instructions. Emulsified PCR reactions were run on a 96 well plate (Eppendorf, Stevenage, UK) on a G-Storm GS4 thermal cycler (G-Storm, Somerton, Somerset, UK) incubating the plates at 95°C for 10 min followed by 40 cycles of 95°C for 15 sec and 60°C for 60 sec, followed by 10 min incubation at 98°C. The temperature ramp increment was 2.5°C/sec for all steps. Plates were read on a Bio-Rad QX-100 droplet reader using QuantaSoft v1.4.0.99 software (Bio-Rad). At least one negative control wells with no DNA were included in every run. The amount of amplifiable RNase P DNA was quantified from the concentration provided by the software. After quantification, cell line DNA was digested using Hind 3-HF™ restriction enzyme (New England BioLabs, Ipswich, Massachusetts, USA) at 37°C for 1 hour.

### Droplet digital PCR for KRAS mutation detection

PCR reaction mixtures were prepared in a total volume of 20 μL containing: 10 μL 2x Supermix for Probes without dUTP (Bio-Rad), relevant primer probe assays, DNA (variable volume) and nuclease-free water (Life Technologies Corporation, Carlsbad, California, USA). Multiple wells were used if required to analyse the necessary DNA eluate. At least 500 pg equivalent of DNA eluate was analysed in each multiplex assay for each patient. 20 μL of PCR reaction mixture was transferred to a sample well in a disposable droplet generator cassette (Bio-Rad). 70 μL of droplet generation oil (Bio-Rad) was then loaded into the oil well for each channel and the cassette loaded into a QX100 Droplet Generator (Bio-Rad). The droplets were then transferred to a 96-well PCR plate. Emulsified PCR reactions were run on a G-Storm GS4 thermal cycler incubating the plates at 95°C for 10 min followed by 40 cycles of 94°C for 30 sec and 54°C for 60 sec, followed by 10 min incubation at 98°C. The temperature ramp increment was 2.5°C/sec for all steps. Plates were read on a Bio-Rad QX-100 droplet reader using QuantaSoft v1.4.0.99 software from Bio-Rad. At least one negative control well with no DNA was included in every run. Each well was then read using the QX100 Droplet Reader (Bio-Rad) and droplets analysed for emission in the HEX or FAM wavelengths. Two-dimensional amplitude plots displaying measured HEX and FAM amplitude for each droplet show four main droplet populations; droplets containing no amplified DNA, droplets with only mutant DNA and high FAM amplitude, droplets with only wild-type DNA and high HEX amplitude and droplets with both wild-type and mutant amplified DNA and high FAM and HEX amplitude. If multiple copies of wild-type and mutant DNA are contained in the same droplet, droplets with higher FAM and HEX amplitude will be read. These droplets have been excluded from any analyses throughout this study.

### Digital PCR analysis

To assess the mutant allele fraction, the concentration of mutant DNA (copies of mutant DNA per droplet) was estimated from the Poisson distribution. Number of mutant copies per droplet Mmu = -ln (1-(nmu/n)), where nmu = number of droplets positive for mutant FAM probe and n = total number of droplets. The DNA concentration in the reaction was estimated as follows: MDNAconc = -ln (1-(nDNAcon/n)), where nDNAconc = number of droplets positive for mutant FAM probe and/or wild-type HEX probe and n = total number of droplets. The mutant allele fraction = Mmu/ MDNAconc. The measured mutant allele frequency describes the mutant allele fraction expressed as a percentage.

### FFPE tissue DNA access and extraction

Formalin fixed paraffin embedded (FFPE) tumour samples from NSCLC patients surplus to clinical care were collected with written patient consent at The Royal Marsden NHS Foundation Trust. FFPE tissue DNA was extracted (after macrodissection if required to ensure >10% tumour content) using the Qiagen DNA FFPE Tissue Kit (Qiagen) as per manufacturer’s instructions. The eluted DNA was stored at -20°C.

### Ethics statement

All patients provided written consent to this study (ethics approval 13/LO/1389, NRES Committee London-Central), which incorporated surplus somatic DNA from the Cancer Research UK Stratified Medicine Programme (ethics approval 11/EE/0202, NRES Committee East of England).

### Sanger sequencing

The region of interest was amplified using 10 μM forward (5’-TATTATAAGGCCTGCTGAAAATG-3’) and reverse (5’-TTGGATCATATTCGTCCACAA-3’) primers, 10 μL Amplitaq Gold® PCR Master Mix (Life Technologies) and 3 ng of template FFPE—derived DNA or 5ng gDNA. PCR conditions were as per manufacturer’s recommendations. Purified PCR product then underwent a further PCR step using chain-terminating dideoxynucleotides (BigDye® 1.0, Life Technologies) and subsequent DNA sequence was read on an automated sequencer.

### Ion torrent proton sequencing

Sequencing libraries were prepared with the Ion AmpliSeq™ Cancer Hotspot Panel v2 (Life Technologies) using the Ion AmpliSeq Library Preparation protocol with 3-5ng of DNA, according to manufacturer’s instructions. Following barcoding, libraries were quantified using qPCR and diluted to 100 pM. Libraries were templated with the Ion OneTouch2 system (Life Technologies) and sequenced on a PI chip using the Ion PI OT2 200 Kit (Life Technologies), 520 flows and an average amplicon length of 112 bases to a mean depth of x2721307. The sequencing resulted in 45272–11767856 reads per sample.

Ion torrent Variant caller v4.0-r73742 with no Hotspot region and configuration “Germ Line Low Stringency” was used for calling variants. Read counts for all positions were computed using pileup (SAMtools v1.1 [12]) and this data was analysed for possible variants using custom Perl and R scripts. Variants at > 3% reported by both analysis methods and not reported in 1000 Genomes Project database (www.1000genomes.org) were identified as possible somatic mutations. The data was cross referenced against the Cosmic database v70 (cancer.sanger.ac.uk) to identify possible hotspot mutations.

### Statistical analysis

Linear regression was performed using the formula r^2^ = 1-(SS_reg_/SS_tot_) where SS_reg_ refers to the sum of the squares of distances to best-fit linear regression line and SS_tot_ refers to the sum of the squares of vertical distances from the null hypothesis (y = mean of all y values) using GraphPad Prism version 6.0a (GraphPad Software, La Jolla, California, USA).

### KRAS multiplex assay specificity

92 wells of *KRAS* wild-type gDNA were analysed with each multiplex. The limit of detection was set at 0.05% to reflect our measured results with spiked *KRAS* mutant gDNA species (S8 Fig). Individual droplets were clustered according to their HEX amplitude into two groups using the kMeans algorithm. A threshold for mutants was estimated based on 1.5 times the median of the FAM values of those drops that belong to the cluster with higher HEX categories. False positive ‘mutant’ droplets were classified as droplets that were in the lower HEX cluster, but whose FAM value exceeds the threshold to reflect the FAM and HEX amplitudes measured for any of the KRAS mutant species tested for in that multiplex assay. A false positive result was defined as three false positive ‘mutant’ droplets as per the Rare Mutation Detection Best Practices Guidelines [13]. To assess the specificity, we calculated the binomial probability of achieving fewer than three ‘mutant’ droplets per 6000 wild-type droplets for each multiplex assay.

## Results

### *KRAS* mutation duplex ddPCR

**Fig 1:**
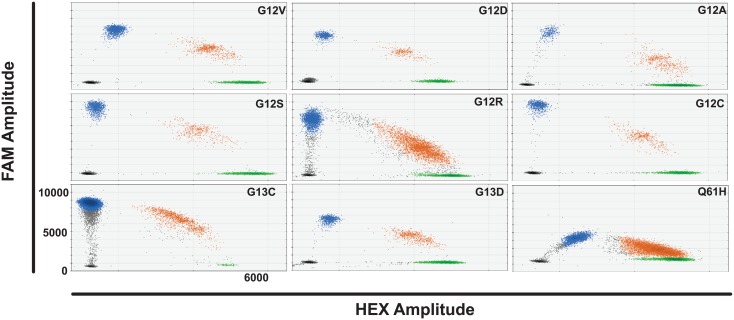
*KRAS* duplex assays at optimal annealing temperature. Droplet populations observed for each duplex assay tested with wild-type and relevant mutant cell line gDNA or oligonucleotide at the optimal annealing temperature e.g. G12V panel top left shows droplet populations seen with WT for G12V assay, G12V assay, NCI-H727 gDNA and NCI-H1975 gDNA present. HEX amplitude is up to 6000 on the x axis and FAM amplitude up to 11000 on the y-axis of each panel. Key: Black drops- empty droplets, blue- mutant DNA FAM positive droplets, green- wild-type DNA HEX positive droplets, brown—wild-type and mutant DNA double positive droplets.

We tested all *KRAS* ddPCR assays separately across an annealing temperature gradient to optimise thermocycling conditions. Each assay was tested with the appropriate gDNA or oligonucleotide alone and then in duplex with wild-type and *KRAS* mutant DNA and both relevant FAM and HEX probes present. Decreasing annealing temperature increased FAM amplitude of the mutant probe to a plateau at 54°C for *KRAS* G12V, D, A, S, R and C and G13C and 13D probes (Fig 1). The *KRAS* Q61H probe had a further minimal increase in FAM amplitude at 53.4°C as compared to 54°C. All probes tested showed good separation of the four different droplet groups at 54°C, allowing clear identification and quantification of different DNA populations.

### *KRAS* multiplex ddPCR assay design

**Fig 2:**
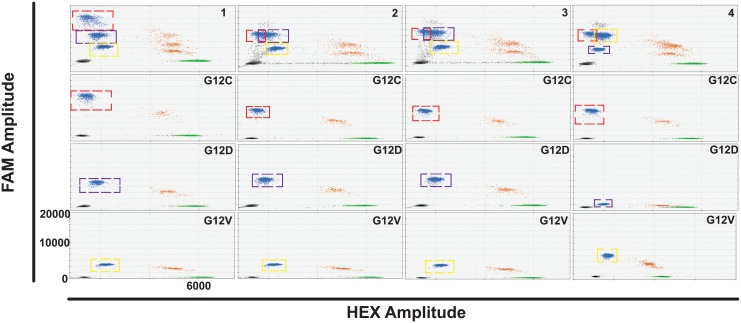
Four different *KRAS* multiplex digital PCR assays combining G12C, G12V and G12D mutant assays and corresponding duplex assays. *KRAS* G12C mutant droplet populations are indicated by a red dashed square, *KRAS* G12D mutant populations by a blue dashed square and *KRAS* G12V mutant populations by a yellow dashed square. Each multiplex assay, combining all relevant FAM and HEX assays, *KRAS* WT gDNA and G12V, D and C mutant gDNA, is shown in the top panel. The corresponding duplex assay for each mutation, using the same FAM and HEX assay concentration with *KRAS* WT DNA and the appropriate *KRAS* mutant DNA present, is shown in the panels below each multiplex assay. Multiplex 1 (top left panel) is an assay combination of 900 nM primers and 500 nM G12C probe, 562.5 nM primers and 312.5 nM G12D probe and 225 nM primers and 125 nM G12V probe with 450 nM primers and 250 nM WT for G12C probe. Multiplex 2 uses the same concentration of G12V and WT for G12C assay as Multiplex 1 but also contains 450 nM primers and 250 nM G12C probe and 675 nM primers and 375 nM G12D probe. Multiplex 3 uses the same concentration of *KRAS* mutant assays as in Multiplex 2 but with the WT for G12V probe assay present, used at the same concentration as the WT for G12C assay in Multiplex 1. Multiplex 4 is an assay combination of the G12C assay as in Multiplex 2 with 225 nM primers and 125 nM G12D probe and 675 nM primers and 375 nM G12V probe with the WT for G12D assay at the same concentration as the WT for G12C assay in Multiplex 1. Key: black- empty droplets, blue- mutant DNA FAM positive droplets, green- wild-type DNA HEX positive droplets, brown—wild-type and mutant DNA double positive droplets. The same scale is used for all panels (HEX amplitude up to 7000 and FAM amplitude up to 22000).

We designed a digital PCR-based multiplex tool to screen for the most common *KRAS* mutations in lung adenocarcinoma; G12C, G12D and G12V (http://www.sanger.co.uk/cosmic). Wild-type probe assays for each mutation were tested in combination with varying concentrations of mutant probe assays, giving rise to mutant droplet populations of varying FAM amplitude (Fig 2). The HEX amplitude of all wild-type probe assays was found to be very similar and so the WT for G12C, WT for G12V and WT for G12D assays were selected as references for all subsequent *KRAS* G12 and G13 mutant assays. Of the different combinations of mutant assays trialled, the multiplex assay which gave best separation of the droplet populations used 900 nM primers and 500 nM G12C probe, 562.5 nM primers and 312.5 nM G12D probe and 225 nM primers and 125 nM G12V probe (Fig 2, top left panel).

The ability to test for multiple *KRAS* mutations would help to detect sub-clonal populations by applying the multiplex approach to clinical samples on which starting material is scarce. To model this, we employed cell-line derived gDNA for the three mutations and tested it with each probe in duplex to ensure specificity (S1 Fig). In the presence of the G12D mutant probe and *KRAS* G12D, V and C mutant DNA, a second mutant population of droplets was identified at lower FAM amplitude to the G12D mutant DNA population (red hatched box, left uppermost panel). This population was not observed when each mutant DNA species was tested in duplex with the G12D probe (left second panel). A similar second mutant population was observed with the G12V mutant probe and all three mutant DNA species as compared with each mutant DNA in duplex (left lower panels). This second population, particularly as seen with the G12D mutant probe, may fall at the expected FAM amplitude of a different *KRAS* mutation in the multiplex assay and lead to false positive mutation detection. To further explore this, FFPE tissue DNA from an individual known to have a *KRAS* 12/13 mutation (F124), as verified by Cobas® testing (Cobas® *KRAS* Mutation Test, Roche Molecular Systems, Inc., Branchburg, NJ, USA) was analysed (S1 Fig, right panels). The multiplex assay identified a mutant droplet population with FAM amplitude that was interpreted as a G12D or G12V mutation. When the sample was tested with each duplex assay individually, no mutant population in either G12V or G12C was observed, whilst a population with a lower FAM amplitude than expected with the G12D mutant assay was observed. On subsequent sequencing and further development of the digital PCR analysis of the tissue DNA, this sample was later identified as *KRAS* G12A mutant (Table 3).

**Table 3:** Detection of *KRAS* mutant clones in FFPE tissue DNA using next generation sequencing (NGS) and *KRAS* multiplex digital PCR assays.

ID	*KRAS* genotype	NGS mutant allele frequency (%)	Digital PCR multiplex mutant allele frequency (%)
S007	G12C	30.4	29.5
S027	G12C	48	53.8
F007	G12D	4	4.6
S002	G12A	32	30
S010	G12C	ND	0.3
	G12A	8.8	8.7
S011	G12V	33.1	32.8
	G12D	ND	0.05
S018	G12F	29	29.5
S030	G13C	17	18.6
S035	G12C	30	19.8
F124	G12A	23	23.7
F130	G12V	28	34.2

Key: ND- not detected.

### Cross-reactivity of *KRAS* probe assays on combination

Due to the close similarities between the DNA sequences of the various *KRAS* mutations, significant cross-reactivity was noted between probes designed for mutations within the same region. This was particularly evident with probes designed for substitutions at the same nucleotide i.e. *KRAS* G12V, D and A and *KRAS* G12S, R and C. The cross-reactivity of the probe assay with ‘mismatched’ DNA carrying a different *KRAS* mutation (S2 and S3 Figs) results in a droplet population of varying FAM and VIC amplitudes relatively close to the empty droplet population and another population close to the true wild-type droplet population. The position of these additional droplet populations dictates the design of multiplex assays to reduce potential misinterpretation of *KRAS* genotypes. Therefore, three multiplex assays were devised to each combine a probe for a mutation at nucleotide position 35, a probe for a mutation at position 34 and a probe for a mutation at either position 37, 38 or 183.

### *KRAS* multiplex ddPCR assay optimisation

Multiplex A is a combination of FAM assays for *KRAS* G13C, G12C and G12V. Several different combinations of mutant probe concentrations were tested and the use of a wild-type probe for G12C and G13C at varying concentrations (S4 Fig). There was significant overlap between the HEX amplitude of the wild-type droplet populations and due to the proximity of the relevant nucleotide bases and the estimated length of the probe, it was felt that 450 nM primers and 250 nM G12C, V or D wild-type probe was adequate for quantification of both G12 and G13 wild-type populations. The optimal multiplex assay (S4 Fig, top left panel) comprised 900 nM primers and 500 nM G13C mutant probe, 450 nM primers and 250 nM G12C probe and 225 nM primers and 125 nM G12V probe. Multiplex B is a combination of FAM assays for *KRAS* G12S, G12D and G13D. Of all the concentrations tested, the optimal combination of mutant probe assays was 675 nM primers and 375 nM G12S probe, 450 nM primers and 250 nM G12D probe and 225 nM primers and 125 nM G13D probe (S5 Fig, top left panel). Multiplex C analyses *KRAS* mutations at G12R, G12A and Q61H. It requires a wild-type probe for both the G12/13 position and the Q61 position for accurate quantification of mutant allele frequency. The assay was optimised with 450 nM primers and 250 nM G12C probe and 900 nM primers and 500 nM Q61H probe as the wild-type assays. The best separation of the mutant droplet populations was achieved using 675 nM primers and 375 nM G12R probe, 450 nM primers and 250 nM G12A probe and 900 nM primers and 500 nM Q61H mutant probe (S6 Fig, top left panel).

**Fig 3:**
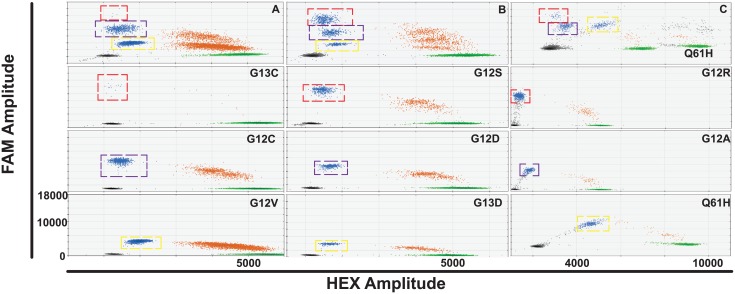
*KRAS* multiplex digital PCR assays A-C and corresponding duplex assays. Multiplex A (top left panel) is an assay combination of 900 nM primers and 500 nM G13C probe (red dashed square), 450 nM primers and 250 nM G12C probe (blue dashed square) and 225 nM primers and 125 nM G12V probe (yellow dashed square). Multiplex B (top middle panel) is an assay combination of 675 nM primers and 375 nM G12S probe (red dashed square), 450 nM primers and 250 nM G12D probe (blue dashed square) and 225 nM primers and 125 nM G13D probe (yellow dashed square). Multiplex C (top right panel) is an assay combination of 675 nM primers and 375 nM G12R probe (red dashed square), 450 nM primers and 250 nM G12A probe (blue dashed square) and 900 nM primers and 500 nM Q61H probe (yellow dashed square). Multiplex C has 900 nM primers and 500 nM Q61H wild-type probe in addition to a G12C wild-type assay. All other wild-type droplet populations shown, except in the Q61H duplex assay, are 450 nM primers and 250 nM G12C wild-type probe. All panels in the left and centre columns show a FAM amplitude up to 18000 and an HEX amplitude up to 6000. Panels in the right column have a FAM amplitude up to 18000 and a HEX amplitude up to 11000.

All optimised multiplex assays (Fig 3) were tested for cross-reactivity with various species of *KRAS* mutant DNA (S7 Fig). There is no overlap between the desired droplet populations and the populations for the other *KRAS* mutant DNA species in all three multiplexes. In addition, the position of droplet populations due to cross-reactivity is highly reproducible and can be used to start to identify which *KRAS* genotype is present in a multiplex assay containing other mutant probes.

### *KRAS* multiplex ddPCR assay characterisation

Decreasing amounts of NCI-H358 (*KRAS* G12C) and A549 (*KRAS* G12S) gDNA were spiked into *KRAS* wild-type gDNA (NCI-H1975) and tested in the appropriate *KRAS* multiplex assay. The limit of detection for *KRAS* G12C mutant DNA in multiplex A was 0.03% and for *KRAS* G12S DNA in multiplex B was 0.045% (S8 Fig, top panels) The measured mutant allele frequency correlated well with decreasing amounts of spiked *KRAS* mutant gDNA in multiplexes A and B (r2 = 0.9992 and 0.0998 respectively), demonstrating the linearity of mutation detection in the multiplex assays. Mutant allele frequency showed little intra-well variability for either multiplex assay and high reproducibility between two different operators on three alternate days for a range of allele frequencies (S8 Fig, middle and bottom panels).

**Fig 4:**
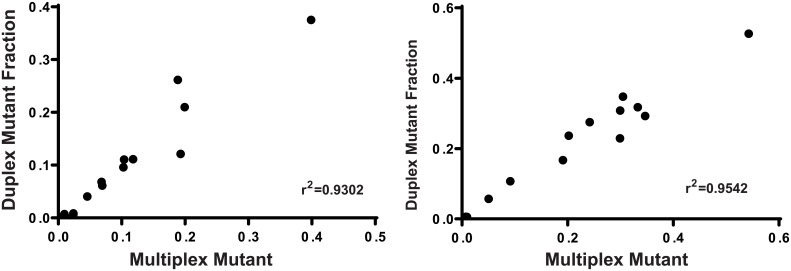
Correlation of mutant DNA fraction detected in multiplex and duplex assays using cell line gDNA or oligonucleotides (left) and in FFPE tissue DNA (right).

Similar *KRAS* allele frequency was observed using multiplex or duplex assays in both cell line DNA (single species *KRAS* mutant gDNA and combinations of *KRAS* mutant gDNA at varying allele frequencies) or oligonucleotides and FFPE tissue DNA (Fig 4; r2 = 0.9302 and 0.9542 respectively).

We analysed multiple wells of NCI-H1975 *KRAS* wild-type gDNA with each of the three *KRAS* multiplex assays and calculated the probability of false positive mutation detection, setting the limit of detection at 0.05% to reflect our measured results with spiked *KRAS* mutant gDNA species (S8 Fig). The specificity of each multiplex is 99.99995%, 99.99857% and 99.85578% for multiplexes A, B and C respectively (S1 Table).

### Comparison of *KRAS* multiplex assay mutant detection with Sanger sequencing

A range of samples containing decreasing amounts of NCI-H358 (*KRAS* G12C) or A549 (*KRAS* G12S) gDNA spiked into *KRAS* wild-type DNA (NCI-H1975) were simultaneously analysed using digital PCR and Sanger sequencing. *KRAS* G12C mutant DNA was detectable using multiplex A down to a mutant allele frequency of 0.2%, whereas the mutant peak on the chromatogram is only visible at a mutant allele frequency of 31.5% and not below 10% (S9 Fig, top panels). *KRAS* G12S mutant DNA remains detectable by digital PCR using multiplex B at a mutant allele frequency of 0.1%, but is only visible using Sanger sequencing at 17%. (S9 Fig, lower panels).

### Detection of *KRAS* mutations in FFPE tissue DNA

**Fig 5:**
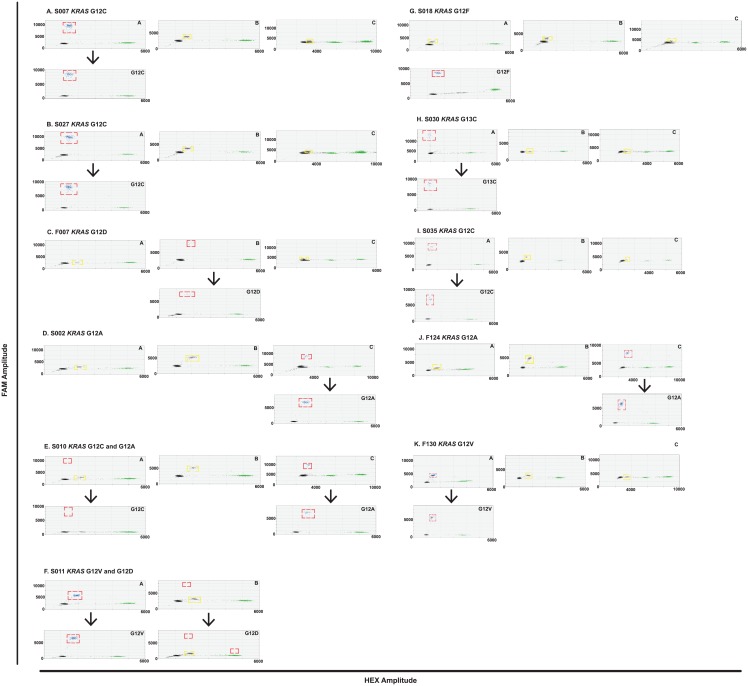
*KRAS* mutant FFPE tissue DNA analysis using multiplex and duplex assays to detect *KRAS* mutant clones. All samples, except for S011, were analysed with multiplexes A, B and C (upper panels) and the *KRAS* mutation detected was subsequently confirmed with the appropriate duplex assay (lower panels). Mutant DNA droplet populations are highlighted with a red dashed square. Droplet populations caused by cross-reactivity with a *KRAS* mutant DNA species not present in the multiplex are indicated by a yellow dashed square.

**Fig 6:**
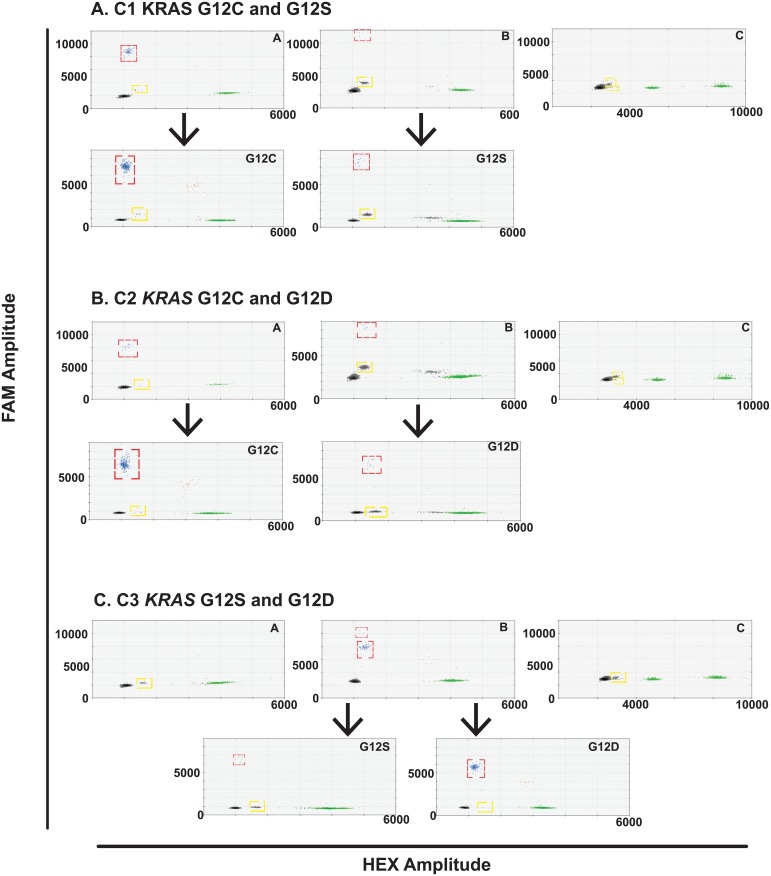
*KRAS* mutant biclonal gDNA analysis using multiplex and duplex assays to detect *KRAS* mutant clones. All samples were analysed with multiplexes A, B and C (upper panels) and the *KRAS* mutation detected was subsequently confirmed with the appropriate duplex assay (lower panels). Mutant DNA droplet populations are highlighted with a red dashed square. Droplet populations caused by cross-reactivity with a *KRAS* mutant DNA species not present in the multiplex are indicated by a yellow dashed square.

FFPE tissue DNA extracted from 11 cases of advanced *KRAS* mutant NSCLC (S2 Table) was analysed using each multiplex assay. The presence of a *KRAS* 12/13 mutation was known from prior Cobas® testing (Cobas® *KRAS* Mutation Test, Roche) but investigators were blinded to the specific *KRAS* genotype. At least one *KRAS* mutation was detected in each case and two cases had two detectable *KRAS* clones (Fig 5). Mutations were confirmed with the appropriate duplex assay. The low frequency G12D mutation in case S011 may represent FFPE artefact due to the frequent deamination of guanine nucleotides during the tissue preservation process [14]. The G12F mutation in S018 was identified by subsequent sequencing of the tissue sample after observation of the cross-reactivity droplet population in all multiplex assays. In addition, two species of KRAS mutant gDNA were spiked into a background of *KRAS* wild-type DNA (NCI-H1975) gDNA to create three samples containing a major and minor KRAS clone; C1 is a combination of NCI-H358 (*KRAS* G12C) and A549 (*KRAS* G12S) gDNA, C2 is a combination of NCI-H358 (*KRAS* G12C) and A427 (*KRAS* G12D) gDNA and C3 is a combination of A427 (*KRAS* G12D) and A549 (*KRAS* G12S) gDNA. All KRAS species were detectable using the KRAS multiplex assays, including the minor clones down to a mutant allele frequency of 0.3% measured for the *KRAS* G12D mutant DNA in sample C3 (Fig 6).

### Comparison of *KRAS* multiplex assay mutant detection with next generation sequencing

**Fig 7:**
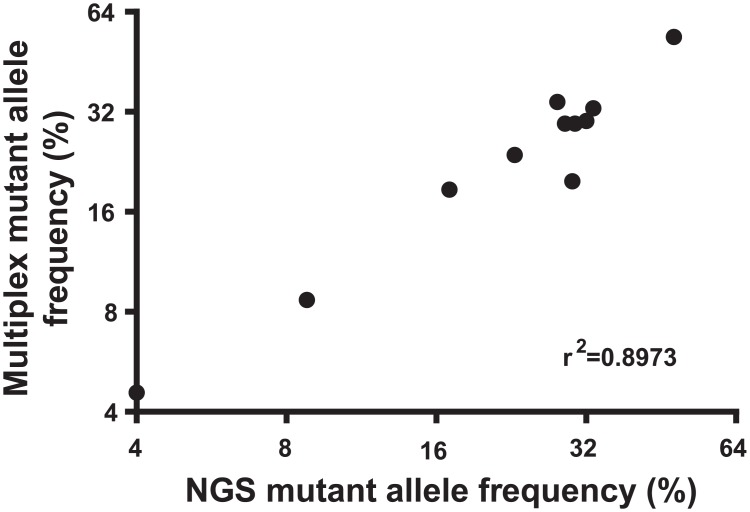
Correlation of NGS *KRAS* mutant allele frequency with digital PCR *KRAS* mutant allele frequency detected in the appropriate multiplex assay for FFPE tissue DNA and cell line gDNA samples.

FFPE tissue DNA from all 11 cases and gDNA from the 3 biclonal cell line DNA samples were sequenced using the Ion Ampliseq Cancer Hotspot Panel v2 on the Ion Proton^TM^ platform (Life Technologies) to analyse the *KRAS* 12 and 13 loci. Next generation sequencing could not detect KRAS mutant allele frequencies below 4% in tissue DNA and none of the minor clones below 2% were detectable in the biclonal gDNA samples in either of the duplicate library preparations (Tables 3 and 4). The frequency of *KRAS* mutant alleles that were detectable on next generation sequencing correlate well with those measured in the KRAS multiplex digital PCR assays (Fig 7, r2 = 0.8973).

**Table 4:** Detection of *KRAS* mutant clones in biclonal gDNA samples using next generation sequencing (NGS) and *KRAS* multiplex digital PCR assays. Two individual library preparations were analysed for each sample.

ID	*KRAS* genotype	Library 1 NGS mutant allele frequency (%)	Library 2 NGS mutant allele frequency (%)	Digital PCR mutant allele frequency (%)
C1	G12C	25	23	19.4
	G12S	ND	ND	1.9
C2	G12C	26	26	18.4
	G12D	ND	ND	0.49
C3	G12D	9	10	9.8
	G12D	ND	ND	0.58

Key: ND- not detected.

## Discussion

We have used commercially available primer probe assays for nine common *KRAS* mutations to design and optimize a multiplex assay approach to genotype *KRAS* mutant cancers using limited material using digital droplet PCR. The Bio-Rad QX200 system has been used to analyse copy number variation in a multiplexed fashion with EvaGreen DNA binding dye [15] but this is the first report of *KRAS* mutation detection multiplex assays on the QX100 system that are based on varying concentrations of the primer probe assays.

These multiplex assays permit the identification of the specific *KRAS* genotype and we have observed that this can be deduced from the reproducible droplet populations caused by cross-reactivity in any of the multiplex assays as well as the positive mutant droplet populations at the predicted FAM amplitude. These multiplexes can therefore be used to identify the presence of rarer *KRAS* mutations at the G12/13 and Q61 positions other than those already tested for in the multiplex assays, as demonstrated with case S018.

Sub-clonal mutations can also be detected using this ddPCR approach to *KRAS* mutation detection. In two cases, S010 and S011, two different *KRAS* mutations were detected both in multiplex and duplex. We have noted, however, that all the mutations tested in multiplex B, are G>A nucleotide changes. Low frequency single nucleotide changes as an artifact of the tissue preservation process have been observed [16]. The *KRAS* G12D mutation observed in S011 (mutant allele frequency 0.05%) may therefore be a false positive result. The low frequency *KRAS* G12C mutation in S010, however, is a G>T change and therefore much more likely to be a true result.

We have demonstrated that the multiplex assays are more sensitive than Sanger and next generation sequencing and highly specific. The assays have linearity down to a limit of detection of 0.03–0.045%. Due to the false positive signal seen from FFPE tissue on NGS below mutant allele frequencies of 1.5%[17], the limit of detection is at best 1 mutant *KRAS* molecule in 50 wild-type *KRAS* molecules. The *KRAS* G12S minor clone in the C1 gDNA sample that was measured at 1.9% on multiplex digital PCR, for example, was not detectable on NGS in either of two independent library preparations. Sanger sequencing, in comparison, has a limit of detection of only 1 in 10 and over a range of mutant allele frequencies, a sensitivity of 20%. Digital PCR maintains a very high specificity despite this increased sensitivity and is approaching the 100% specificity seen with NGS [17]. The accuracy of *KRAS* multiplex ddPCR is reflected by the correlation observed with the mutant allele frequency detected by Ion Torrent Proton^TM^ sequencing, an established method of determining this variable. In addition, we have demonstrated the robustness of the multiplex assays across a range of mutant allele frequencies both within the same experiment and between operators on three non-consecutive days.

We have described the combination and optimization of commercially available digital PCR assays to develop novel multiplex assays that allow the accurate detection and discrimination of *KRAS* genotypes in patient-derived material. Multiplexing of assays reduces the amount of clinical material required for testing, which will be particularly important in settings where the proportion of mutant relative to wild-type DNA is likely to be low, such as with circulating free tumour DNA from plasma samples. The three multiplexed assays described here between them will identify the nucleotide changes in 95% of the cancer cases where the *KRAS* gene is mutated.

## Supporting Information

S1 Fig Specificity of *KRAS* multiplex assay in presence of various mutant *KRAS* DNA species. Left panels: *KRAS* G12V, G12C and G12D mutant DNA tested with 562.5 nM primers and 312.5 nM G12D FAM probe in one well (top left panel) or in three separate wells (i.e. one well G12V DNA with G12D probe assay etc.). Wells with only one type of *KRAS* mutant DNA are merged (left second panel) to compare with the single well containing all DNA species. *KRAS* G12V, G12C and G12D mutant DNA tested with 225 nM primers and 125 nM G12V FAM probe in one well (left third panel) or in three separate wells (wells merged, left bottom panel). A second droplet population with lower FAM amplitude is detected when more than one species of *KRAS* mutant DNA is present (red dashed box). Right panels: *KRAS* 12/13 FFPE tissue DNA sample (F124) tested with multiplex assay (top right) and then 3 separate duplex assays as indicated. The same scale is used for all panels (HEX amplitude up to 7000 and FAM amplitude up to 11000).
(EPS)

S2 Fig Cross-reactivity of *KRAS* mutant probes with *KRAS* mutation variants. Duplex assays for different *KRAS* mutations (horizontal) with different *KRAS* mutation variants (vertical). More cross-reactivity, indicated by a droplet population near the empty droplets with relatively low FAM and HEX amplitude and a population near the true wild-type population (examples in red dashed square in top right panel), was seen between probes and DNA for the same nucleotide position in exon 2 of *KRAS*. Each panel shows FAM amplitude of up to 10000 and HEX amplitude between 1000 and 6000.
(EPS)

S3 Fig Cross-reactivity of *KRAS* mutant probes with *KRAS* mutation variants (greater magnification). Duplex assays for different *KRAS* mutations (horizontal) with different *KRAS* mutation variants (vertical). More cross-reactivity, indicated by a droplet population near the empty droplets with relatively low FAM and HEX amplitude and a population near the true wild-type population (examples in red dashed square in top right panel), was seen between probes and DNA for the same nucleotide position in exon 2 of *KRAS*. Key: X—omitted panel of duplex assay with correct probe and *KRAS* mutation variant as higher magnification. Each panel shows FAM amplitude of up to 3000 and HEX amplitude between 1000 and 6000.
(EPS)

S4 Fig *KRAS* multiplex digital PCR assays combining G12C, G12V and G13C and corresponding duplex assays. G13C populations are indicated by a red dashed square, G12C populations by a blue dashed square and G12V droplet populations are indicated by a yellow dashed square. Multiplex 1 (top left panel) is an assay combination of 900 nM primers and 500 nM G13C probe, 450 nM primers and 250 nM G12C probe and 225 nM primers and 125 nM G12V probe, with 900 nM primers and 500 nM WT for G12C probe and 450 nM primers and 250 nM WT for G13C probe. Multiplex 2 (centre left) uses the same FAM assay concentrations as in Multiplex 1 with 450 nM primers and 250 nM WT for G12C probe and 900 nM primers and 500 nM WT for G13C probe. Multiplex 3 (centre right) is a combination of 450 nM primers and 250 nM G13C probe and 900 nM primers and 500 nM G12C probe with the same G12V and WT G12C and G13C assays as in Multiplex 1. Multiple 4 uses the same FAM assays concentrations as Multiplex 3 with the WT for G12C and G13C assay concentrations as in Multiplex 2. Each panel shows a FAM amplitude up to 18000 and a HEX amplitude up to 12000.
(EPS)

S5 Fig *KRAS* multiplex digital PCR assays combining G12S, G12D and G13D and corresponding duplex assays. G12S droplet populations are indicated by a red dashed square, G12D populations by a blue dashed square, and G13D populations by a yellow dashed square. Multiplex 1 (top left panel) is an assay combination of 675 nM primers and 375 nM G12S probe, 450 nM primers and 250 nM G12D probe and 225 nM primers and 125 nM G13D probe with 225 nM primers and 125 nM WT for G12C probe and 450 nM primers and 250 nM WT for G13D probe. Multiplex 2 (top middle panel) is a FAM assay combination of 225 nM primers and 125 nM G12S probe, 675 nM primers and 375 nM G12D probe and 450 nM primers and 250 nM G13D probe with the same WT for G12C and G13D assay concentrations as in Multiplex 1. Multiplex 3 (top right panel) is a combination of, 450 nM primers and 250 nM G12S probe, 675 nM primers and 375 nM G12D probe and the same FAM 13D assay concentration as in Multiplex 1. WT assay concentrations are 450 nM primers and 250 nM WT for G12C probe and 225 nM primers and 125 nM WT for G13D probe. Each panel shows a FAM amplitude up to 18000 and a HEX amplitude up to 9000.
(EPS)

S6 Fig *KRAS* multiplex digital PCR assays combining G12R, G12A and Q61H and corresponding duplex assays. G12R droplet populations are indicated by a red dashed square, G12A populations by a blue dashed square and Q61H populations by a yellow dashed square. Multiplex 1 (top left panel) is an assay combination of 675 nM primers and 375 nM G12R probe, 450 nM primers and 250 nM G12A probe and 900 nM primers and 500 nM Q61H probe with 450 nM primers and 250 nM WT for G12C probe and 900 nM primers and 500 nM WT for Q61H probe. Multiplexes 2–4 use the same WT assay concentrations. Multiplex 2 (centre left panel) is a FAM assay combination of 450 nM primers and 250 nM G12R probe and 675 nM primers and 375 nM G12A probe with the same FAM Q61H assay concentration as in Multiplex 1. Multiplex 3 (centre right panel) is an assay combination of 900 nM primers and 500 nM G12R probe, 225 nM primers and 125 nM G12A probe and 450 nM primers and 250 nM Q61H probe. Multiplex 4 (top right panel) is an assay combination of 450 nM primers and 250 nM G12A probe and 225 nM primers and 125 nM Q61H probe with the same FAM G12R assay as in Multiplex 3. Each panel shows a FAM amplitude up to 16000 and a HEX amplitude up to 11000.
(EPS)

S7 Fig Cross-reactivity of *KRAS* multiplex assays with *KRAS* mutation variants. Droplet populations seen with the addition of cell line DNA or oligonucleotides containing different *KRAS* mutation variants and analysed with multiplexes A, B and C. Multiplex A is an assay combination of G13C, G12C and G12V. Multiplex B is an assay combination of G12S, G12D and G13D. Multiplex C is an assay combination of G12R, G12A and Q61H. Panel A1 –multiplex A with *KRAS* G12V, C, D, S and R DNA; panel A2 –multiplex A with *KRAS* G12A DNA; panel A3- multiplex A with *KRAS* G13C and G13D DNA. Panel B1 –multiplex B with *KRAS* G12V, C, D, S and R DNA; panel B2 –multiplex B with *KRAS* G12A DNA; panel B3- multiplex B with *KRAS* G13C and G13D DNA. Panel C—multiplex C with *KRAS* G12V, C, D, S, R, A, 13C, 13D and Q61H DNA. Panels A1-3 and B1-3 show a FAM amplitude up to 18000 and an HEX amplitude up to 7000. Panel C has a FAM amplitude up to 18000 and a HEX amplitude up to 10000. Key: * = *KRAS* G12V mutant DNA, ¶ = G12D, ⌃ = G12C, ★ = G12S, ♦ = G12R, • = G12A, § = G13C, ■ = G13D; black- empty droplets, blue- mutant DNA FAM positive droplets, green- wild-type DNA HEX positive droplets, brown—wild-type and mutant DNA double positive droplets.
(EPS)

S8 Fig KRAS multiplex assay performance. Left panels: Multiplex A tested with decreasing amounts of *KRAS* mutant G12C DNA spiked in against a *KRAS* wild-type DNA background; right panels: Multiplex B tested with decreasing amounts of *KRAS* mutant G12S DNA against a *KRAS* wild-type DNA background. Top panels: Correlation of amount of input *KRAS* mutant DNA with measured mutant allele frequency. Middle panels: Mutant allele frequency per well as compared with mean mutant allele frequency across all wells for a range of allele frequencies (0.05–4% multiplex A and *KRAS* G12C, 0.08–3% multiplex B and *KRAS* G12S). Bottom panels: Mutant allele frequency measured on three non-adjacent days by two different operators across a range of amounts of spiked *KRAS* mutant DNA. Key: Triangle = Day 1, square = Day 2, circle = Day 3.
(EPS)

S9 Fig Comparison of *KRAS* mutant DNA detection in *KRAS* multiplex assays A and B compared with Sanger sequencing of gDNA samples. *KRAS* G12C mutant DNA samples at allele frequencies ranging from 0.2–31.5% analysed by digital PCR in *KRAS* multiplex assay A (upper panels) or Sanger sequencing (upper chromatograms). *KRAS* G12S mutant DNA samples at allele frequencies ranging from 0.1–17% analysed by digital PCR in *KRAS* multiplex assay B (lower panels) or Sanger sequencing (lower chromatograms). All panels in the upper row show a FAM amplitude up to 10000 and a HEX amplitude up to 6000. Panels in the lower row have a FAM amplitude up to 14000 and a HEX amplitude up to 11000. Key: black- empty droplets, blue- mutant DNA FAM positive droplets, green- wild-type DNA HEX positive droplets, brown—wild-type and mutant DNA double positive droplets; black arrow—mutant nucleotide not discernible from background signal on Sanger sequencing, red arrow—mutant thymine nucleotide detectable using Sanger sequencing, green arrow—mutant adenine nucleotide detectable using Sanger sequencing.
(EPS)

S1 Table *KRAS* multiplex assay specificity. (DOC)

S2 Table Clinical characteristics of 11 patients with lung adenocarcinoma included in the FFPE tissue analysis. Key: F- former smoker, S- smoker, N- never smoker, Unk- unknown.
(DOC)
