# Supplementary material for: Efficient Genotyping of KRAS Mutant Non-Small Cell Lung Cancer Using a Multiplexed Droplet Digital PCR Approach
Source: PLoS One. 2015 Sep 28;10(9):e0139074. doi: 10.1371/journal.pone.0139074 (PMC4586384; doi:10.1371/journal.pone.0139074)
Supplement: S1 Table — (DOC) [file pone.0139074.s010.doc]

| **KRAS multiplex assay** | **Number of positive events** | **Number of wild-type events** | **Binomial specificity (%)** |
| --- | --- | --- | --- |
| A | 4 | 416707 | 99.99995 |
| B | 9 | 401755 | 99.85578 |
| C | 21 | 228460 | 99.99857 |
