# Supplementary material for: Efficient Genotyping of KRAS Mutant Non-Small Cell Lung Cancer Using a Multiplexed Droplet Digital PCR Approach
Source: PLoS One. 2015 Sep 28;10(9):e0139074. doi: 10.1371/journal.pone.0139074 (PMC4586384; doi:10.1371/journal.pone.0139074)
Supplement: S2 Table — Key: F- former smoker, S- smoker, N- never smoker, Unk- unknown. (DOC) [file pone.0139074.s011.doc]

| **ID** | **Age** | **Gender** | **Smoking status** | **Tobacco exposure (pack years)** | **Subtype** | **Stage** |
| --- | --- | --- | --- | --- | --- | --- |
| S007 | 61 | F | F | 20 | Adenocarcinoma | IV |
| S027 | 62 | F | S | 31 | Adenocarcinoma | IV |
| S002 | 68 | F | S | Unk | Adenocarcinoma | IV |
| S010 | 74 | F | S | 58 | Adenocarcinoma | Unk |
| S011 | 66 | M | F | 37 | Adenocarcinoma | IIIa |
| S018 | 60 | M | F | 40 | Adenocarcinoma | IV |
| S030 | 63 | M | F | 97 | Adenocarcinoma | IV |
| S035 | 73 | M | F | 55 | Adenocarcinoma | IV |
| F124 | 70 | F | N | 0 | Adenocarcinoma | IV |
| F130 | 57 | F | F | 60 | Adenocarcinoma | IIb |
